# Serum and synovial fluid lipidomic profiles predict obesity-associated osteoarthritis, synovitis, and wound repair

**DOI:** 10.1038/srep44315

**Published:** 2017-03-20

**Authors:** Chia-Lung Wu, Kelly A. Kimmerling, Dianne Little, Farshid Guilak

**Affiliations:** 1 Department of Orthopaedic Surgery, Washington University, St. Louis MO, 63110, USA; 2 Shriners Hospitals for Children – St. Louis, St. Louis MO, 63110, USA; 3 Departments of Basic Medical Sciences and Biomedical Engineering, Purdue University, West Lafayette, IN, 47907, USA.

## Abstract

High-fat diet-induced obesity is a major risk factor for osteoarthritis (OA) and diminished wound healing. The objective of this study was to determine the associations among serum and synovial fluid lipid levels with OA, synovitis, adipokine levels, and wound healing in a pre-clinical obese mouse model of OA. Male C57BL/6 J mice were fed either a low-fat (10% kcal) or one of three high-fat (HF, 60% kcal) diets rich in saturated fatty acids (SFAs), ω-6 or ω-3 polyunsaturated FAs (PUFAs). OA was induced by destabilization of the medial meniscus. Mice also received an ear punch for evaluating wound healing. Serum and synovial fluid were collected for lipidomic and adipokine analyses. We demonstrated that the serum levels of ω-3 PUFAs were negatively correlated with OA and wound size, but positively correlated with adiponectin levels. In contrast, most ω-6 PUFAs exhibited positive correlations with OA, impaired healing, and inflammatory adipokines. Interestingly, levels of pentadecylic acid (C15:0, an odd-chain SFA) and palmitoleic acid were inversely correlated with joint degradation. This study extends our understanding of the links of FAs with OA, synovitis and wound healing, and reports newly identified serum and synovial fluid FAs as predictive biomarkers of OA in obesity.

Osteoarthritis (OA) is a progressive joint disease characterized by degradation of articular cartilage, inflammation of synovium, osteophyte formation, and subchondral bone remodeling^1^,^2^. It is estimated that over 27 million people suffer from OA in the U.S., and this number could double by 2030^3^. Clinical symptoms include joint pain, stiffness, and disability; however, there are currently no disease-modifying drugs available for OA, and few therapies for end-stage OA other than total joint replacement.

One of the impediments to developing new therapies for OA has been the lack of effective and minimally-invasive methods to predict, diagnose, and monitor disease progression. In this regard, there have been expanded efforts in recent years to identify biomarkers of OA. According to the Biomarkers Definitions Working Group, a biomarker is an indicator of normal biological processes, pathogenic processes, or pharmacologic responses to a therapeutic intervention^4^. While a number of biomarker studies have focused on the release of cartilage matrix proteins such as collagens, proteoglycans, or cartilage oligomeric matrix protein in the serum and synovial fluid^5^,^6^,^7^, mounting evidence suggests that OA is more than just cartilage wear and tear, as both systemic and local intra-articular metabolic factors such as inflammation appear to play a pivotal role in joint degeneration^8^,^9^. Thus, in addition to matrix degradation products, inflammatory cytokines and adipokines may also have potential as OA biomarkers that are related to disease mechanisms^10^,^11^.

Major risk factors for OA include aging and joint injury, although it has been argued that obesity may be the primary preventable risk factor for OA^12^ as well as for impaired wound healing. Several studies have demonstrated that imbalanced intake of dietary saturated fatty acids (SFAs) and ω-6 polyunsaturated fatty acids (ω-6 PUFAs) may be associated with elevated systemic inflammation in obesity, while ω-3 PUFAs are generally believed to be anti-inflammatory FAs^13^,^14^. Using a high-fat (HF) diet-induced obesity mouse model, we recently demonstrated that mice fed a HF diet rich in ω-3 PUFAs had mitigated joint degeneration with superior ear wound healing compared to mice fed HF diets rich in either pro-inflammatory SFAs or ω-6 PUFAs^15^. Furthermore, bone marrow-derived mesenchymal stem cells and adipose stem cells had altered proliferation and differentiation capacity when cultured with SFA *in vitro*, providing a potential mechanism for impaired wound healing in obesity^16^. Studies also suggest that in response to a HF environment, chondrocytes change their metabolic behaviors and increase pro-inflammatory cytokine production^17^,^18^.

As they are metabolized, dietary FAs are incorporated into cell membranes, stored in the form of higher-order lipids such as triglycerides and phospholipids, converted into lipid mediators including oxylipins, and/or simply exist as free form in the serum^19^. For example, ω-3 PUFAs give rise to anti-inflammatory oxylipins such as protectins and resolvins, whereas ω-6 PUFAs produce pro-inflammatory oxylipins including numerous prostaglandins and leukotrienes^20^. In addition, it has been demonstrated that the surface of cartilage is covered by a layer of phospholipids, and this phospholipid layer serves as boundary lubricant during joint loading^21^. Therefore, changes in the composition of this lubrication layer due to either injury or dietary FAs may impact the tribological function of the articular joint, potentially leading to the onset of OA^22^.

Furthermore, several clinical studies have revealed correlations between various lipid classes in serum or synovial fluid with OA disease stage. For instance, Oliviero and co-workers observed increased levels of serum total triglycerides and cholesterols in OA patients compared to those in healthy subjects^23^. Another study supporting this result showed that the incidence of bone marrow lesions, another risk factor driving OA development, is significantly associated with levels of serum triglycerides and cholesterol in middle-aged women^24^.

Despite the fact that these studies have greatly extended our knowledge in understanding the link between various lipid species and OA, the wide range of age and various dietary habits of the subjects, however, may complicate interpretation of clinical study results. Furthermore, there is a paucity of metabolomics or lipidomics studies investigating the relationships between lipid species, joint inflammation (particularly synovitis), and wound healing in obesity. Of particular interest are recent findings showing a positive correlation between ear wound healing, knee cartilage regeneration, and protection from OA^25^. In this regard, the goal of the current study was to examine the relationship between serum and synovial fluid lipidomic panels of FAs with OA severity, joint synovitis and wound regeneration capacity in a previously reported obese animal model of OA in which the mice were fed prescribed HF diets rich in various dietary FAs.

## Materials and Methods

### Animals

All procedures were performed in accordance with a protocol approved by the Animal Studies Committee at Duke University and Washington University School of Medicine. Beginning at 4 weeks of age, male C57BL/6 J mice were fed either a control low-fat (10% kcal fat) or one of three HF (60% kcal fat) diets rich in SFAs, ω-6 PUFAs, or ω-3 PUFAs for 24 weeks^15^. At 16 weeks of age, mice underwent surgery for destabilization of the medial meniscus (DMM) to induce knee OA in the left hind limb. All mice also received an ear punch (∅ = 1.5 mm) for the evaluation of the effect of dietary FAs on wound healing capacity. Upon sacrifice, OA severity, synovitis, and ear repair were determined by histologic grading and were reported previously^15^.

### Metabolite Analysis

Serum and synovial fluid samples were collected at the end of the study as previously described^15^. Serum samples (control diet, n = 7; SFA diet, n = 9; ω-6 diet, n = 10; ω-3 diet, n = 9) and synovial samples (n = 6 for each diet) were submitted to Metabolon (TrueMass^®^ Fatty Acid Metabolism Panel) for FA analyses. Briefly, lipids were extracted in chloroform-methanol and trans-esterified in sulfuric acid/methanol to create fatty acid methyl esters (FAME). FAME was used for gas chromatography. The absolute concentration of each fatty acid in the original sample was determined by comparing the peak area to that of the internal standard. A total of 28 FAs and 4 plasmalogen derivatives were analyzed (Supplementary Table 1).

Using algorithms developed by Metabolon in the Surveyor Tool©, comparisons were made of FA metabolism enzyme signatures or pathways including lipogenesis, plasmalogens, ω-3: ω-6 ratio, stearoyl-CoA desaturase (SCD), Δ-5 and Δ-6 desaturase (D5D and D6D) in serum and synovial fluid from DMM- and non-operated joints. Data was reported as a percentage change for the overall numerator dataset when compared to the denominator dataset.

### Statistical analysis

In the current study, the absolute concentration (calculated as nMole per gram material) and normalized concentration (calculated as molar percentage of total fatty acids) were each evaluated. Using the right non-operated joint as a contralateral control, the percentage change of synovial FAs due to DMM in either molar fraction or in nMole/gram material was also computed. Bivariate regression analyses were used to evaluate the associations among FAs, adipokines, OA, synovitis, and wound healing. Analyses were performed using IBM SPSS Statistics version 23, with significance reported at the 95% confidence level.

## Results

### Serum FA levels

**Figure 1:**
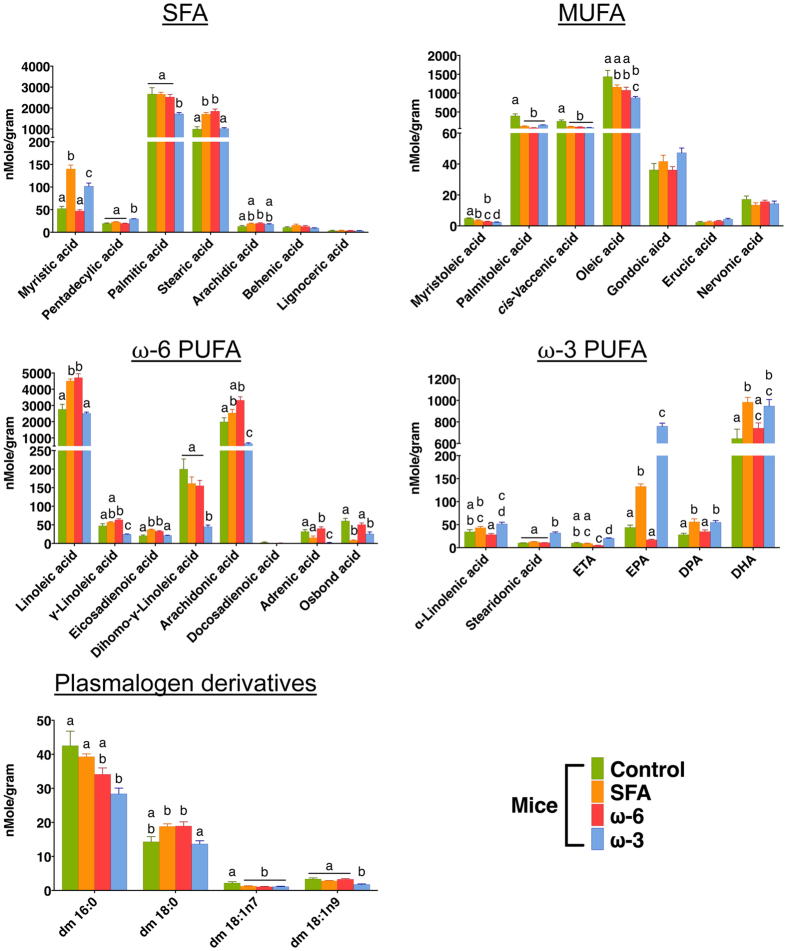
Absolute concentrations of serum lipid species (nMole/gram material). The ω-3 mice had relatively low absolute concentration of serum SFAs, ω-6 PUFAs, and plasmalogen derivatives, but significantly higher absolute concentration of serum eicosapentaenoic acid (EPA), compared to the mice fed other HF diets. One-way ANOVA with Tukey’s post-hoc was performed to evaluate the effect of diet on each lipid concentration. Different letters are significantly different from each other (p < 0.05).

Regardless of the diets and the units used for the FAs, the predominant FA species in serum were palmitic and stearic acid among SFAs, palmitoleic, *cis-*vaccenic, and oleic acid among monounsaturated fatty acids (MUFAs), linoleic and arachidonic acid among ω-6 PUFAs, docosahexaenoic acid (DHA) among ω-3 PUFAs, and plasmalogen palmitic (dm16:0) among plasmalogen derivatives (Fig. 1 and Supplementary Figure 1).

The ω-3 mice had relatively low absolute concentrations (nMole per gram material, nMole/gram) of serum SFAs, ω-6 PUFAs, and plasmalogen derivatives, but as expected, significantly higher absolute concentrations of serum ω-3 PUFAs such as stearidonic acid, eicosatetraenoic acid (ETA), and eicosapentaenoic acid (EPA) compared to the mice fed other HF diets (Fig. 1). Interestingly, ω-3 mice had comparable absolute concentration of serum DHA to the mice fed the other HF diets, although serum DHA levels were the lowest in ω-6 mice.

When the normalized concentration (molar percentage of total fatty acids, mol%) was expressed, the ω-3 mice still exhibited relatively low normalized concentrations of ω-6 PUFAs, but high normalized concentrations of MUFAs and ω-3 PUFAs (Supplementary Figure 1). Furthermore, normalized concentration of serum DHA of ω-3 mice was significantly higher than that of the mice treated with other diets.

### Synovial fluid FA levels

**Figure 2:**
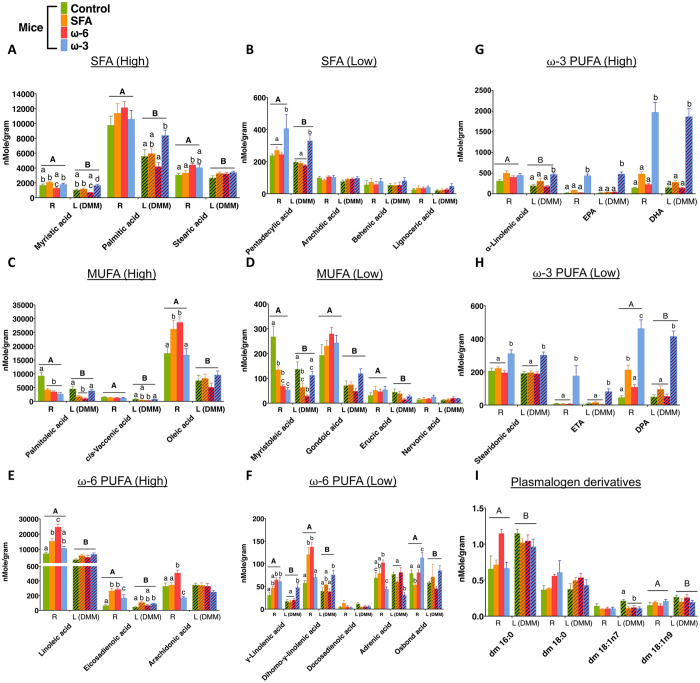
Absolute concentrations of the lipid species in the synovial fluid from non-operated right (R) joint and from DMM-operated left (L) joint. (**A**,**C**,**E**,**G**) are FA plots with higher concentrations, while (**B**,**D**,**F**,**H**) are FA plots with lower concentrations. The ω-3 mice had significantly higher absolute concentrations of pentadecylic acid and most ω-3 PUFAs in synovial fluid from both non-operated and DMM joints. Two-way repeated measures ANOVA with Tukey’s post-hoc was performed to evaluate the effect of diet and surgery on each lipid concentration. Different letters are significantly different from each other (p < 0.05).

**Figure 3:**
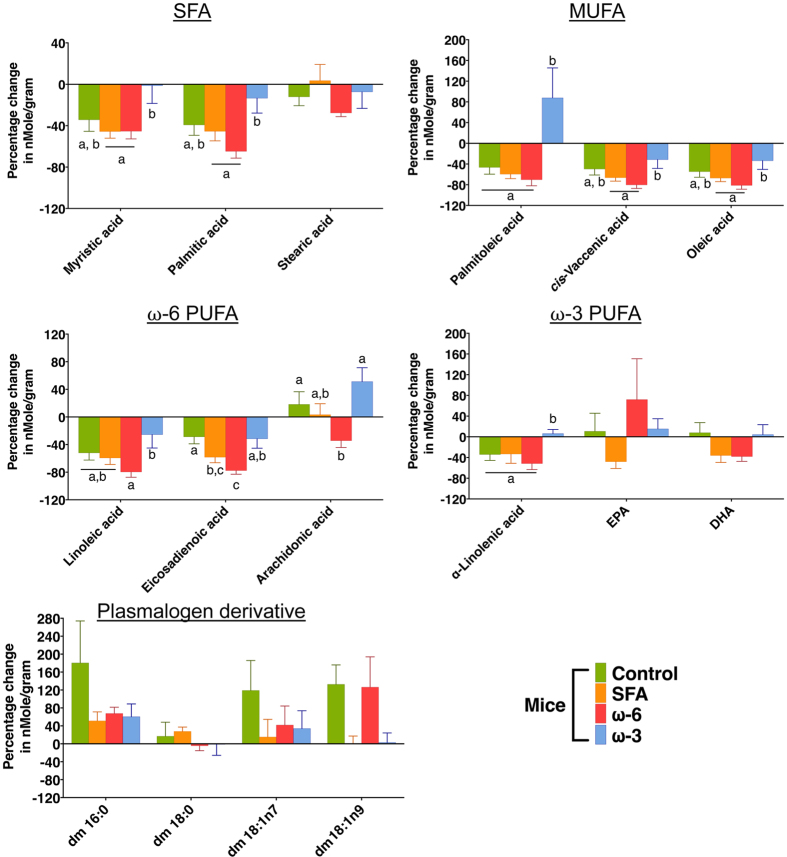
The percentage change of absolute concentration for major synovial FAs prior- and post-DMM surgery. Surgery significantly decreased the absolute concentrations of most lipid species in synovial fluid in the operated joints. In general, ω-6 mice tended to have larger changes in the absolute concentration of synovial fluid FAs post-surgery, while ω-3 mice appeared to have less alterations in lipid composition of synovial fluid. One-way ANOVA with Tukey’s post-hoc was performed to evaluate the effect of diet on each lipid concentration. Different letters are significantly different from each other (p < 0.05).

The absolute and normalized concentrations of synovial fluid FAs are presented in Fig. 2 and Supplementary Figure 2, respectively. The absolute concentrations of the synovial fluid FAs were significantly greater than their counterparts in serum. Furthermore, regardless of the diets and the units used for the FAs, synovial fluid from both non-operated and DMM joints had high levels of myristic acid, palmitic acid and stearic acid among SFAs, high levels of palmitoleic acid, *cis*-vaccenic acid, and oleic acid among MUFAs, high levels of linoleic acid, eicosadienoic acid and arachidonic acid among ω-6 PUFAs, and high levels of α-linolenic acid, EPA and DHA among ω-3 PUFAs. The ω-3 mice exhibited significantly greater absolute concentrations of pentadecylic acid (an odd-chain FA) and most ω-3 PUFAs in synovial fluid from both non-operated and DMM joints. Figure 3 shows the percentage change of absolute concentration for major synovial FAs prior- and post-DMM surgery, using the right non-operated joint as a contralateral control joint for the surgery (i.e. as prior-DMM surgery). We observed that DMM surgery significantly decreased the absolute concentrations of most synovial fluid FAs in the operated joints. In general, ω-6 mice tended to have larger changes in the absolute concentration of synovial fluid FAs post-surgery, while ω-3 mice appeared to have less alteration in synovial fluid FAs among the obese mice. Interestingly, ω-3 mice had increased absolute concentration of synovial palmitoleic acid after DMM surgery.

When the normalized concentrations and percentage change in mole fraction for each synovial FA were calculated (Supplementary Figure 2 and Supplementary Figure 3), we observed that DMM surgery actually led to increased normalized concentrations of SFAs, arachidonic acid, ω-3 PUFAs, and plasmalogen derivatives in the synovial fluid of the operated joint. DMM surgery, however, decreased normalized concentrations of MUFAs in the operated joint, except that palmitoleic acid was increased in ω-3 mice. Similar to the percentage change in absolute concentration, ω-6 mice exhibited substantial changes in the normalized concentrations of synovial fluid FAs post-surgery, while ω-3 mice appeared to have less modification in synovial fluid FA levels among the obese mice (Supplementary Figure 3).

### OA, synovitis, ear wound healing, and serum adipokines

The results of OA severity, joint synovitis, ear wound size, and serum adipokine levels (insulin, leptin, adiponectin, and resistin) were reported in our recently published study^15^. Briefly, the mice fed ω-3 PUFAs demonstrated reduced joint degradation and improved ear wound repair. The ω-3 mice also demonstrated decreased serum insulin, leptin and resistin, but significantly higher serum adipokine levels compared to the mice treated with other HF diets. The data sets of OA score, synovitis score, ear wound area, and adipokine concentrations from the aforementioned study were used to perform linear regression analyses to investigate their associations with serum and synovial fluid FAs.

### Correlation of serum FAs with OA, synovitis, and ear wound area

When absolute concentration was used as the unit for the regression models, 5 out 32 serum lipids measured in the study showed correlations with both OA severity and joint synovitis at the same time: stearic acid, linoleic acid, γ-linolenic acid, eicosadienoic acid, and ETA (Table 1). Among these five serum FAs, stearic acid and all three ω-6 PUFAs were positively correlated with the joint disease markers, while ETA (a ω-3 PUFA) showed a negative correlation. Interestingly, pentadecylic acid, a SFA, exhibited significant negative association with OA score and wound area. Among MUFAs, only palmitoleic acid and erucic acid were negatively associated with OA, and nervonic acid was negatively correlated with joint synovitis. In addition, two serum SFAs (myristic acid and pentadecylic acid) and four serum ω-3 PUFAs (α-linolenic acid, stearidonic acid, ETA, and EPA) were negatively correlated with ear wound size, but none of the ω-6 PUFAs were associated with ear wound healing. Notably, the serum ω-3: ω-6 ratio significantly correlated with OA and wound regeneration, and trended towards correlation with joint synovitis.

When normalized concentrations of serum FAs were used to perform bivariate correlation with OA and synovitis score, only three serum FAs (palmitoleic acid, linoleic acid, and ETA) were correlated with these two disease scores at the same time (Supplementary Table 2). However, a greater correlation between serum ω-6 PUFAs and ear wound area was observed; most of them were positively associated with ear wound size. Interestingly, pentadecylic acid still exhibited a negative correlation with OA severity and ear wound area and trended toward a negative association with synovitis when normalized concentrations were used in the regression model.

### Correlation of absolute concentration of serum FAs with serum adipokines

Pro-inflammatory factors for metabolic disorders such as serum insulin, leptin, and resistin were positively correlated with serum stearic acid and γ-linolenic acid simultaneously (Table 2). Most ω-6 PUFAs and plasmalogen derivatives in the current study also exhibited positive correlation with serum insulin and leptin concentrations. All measured ω-3 PUFAs exhibited negative correlation with serum leptin, while EPA and DHA had positive correlation with serum adiponectin. Furthermore, serum ω-3: ω-6 ratio negatively associated with serum insulin, leptin, and resistin, but was positively correlated with adiponectin.

### Correlation of synovial FAs with OA and synovitis

When absolute concentration was used as the unit and applied to the regression models, twelve synovial fluid FAs from the DMM joints showed significant associations with OA severity; however, none of the synovial FAs measured from the DMM joints correlated with synovitis (Table 3). Notably, among these 12 synovial FAs, only docosadienoic acid had a positive association with OA, while the others, including all the SFAs, exhibited negative association with OA severity. Furthermore, no relationship was observed for the ω-3: ω-6 PUFAs ratio from the synovial fluid with either OA severity or synovitis.

When normalized concentration was used as the unit for the regression models, seven synovial fluid FAs and three plasmalogen derivatives from the DMM joints showed significant associations with OA severity, while nine synovial fluid FAs from the DMM joints correlated with synovitis score (Supplementary Table 3). Among them, behenic acid, palmitoleic acid, docosadienoic acid, and adrenic acid were associated with both OA and synovitis.

### Correlation of serum FAs with synovial FAs

Not all the serum FAs correlated with their counterparts in the synovial fluid. Furthermore, serum FAs correlated better with synovial FAs from right non-operated joints than those from the left DMM-operated joints, independent of the unit used (Supplementary Table 4 and Supplementary Table 5).

### The effect of diets and injury on metabolic pathways

The effect of the injury on the metabolic signatures in the synovial fluid within each diet group was compared. For control mice, there was no difference in FA metabolism signatures between DMM and non-operated limb synovial fluid (Supplementary Figure 4). The SFA mice and ω-6 mice had increased lipogenesis, D5D products, and plasmalogens, but decreased SCD products in the synovial fluid from the DMM joint compared to those from the non-operated joint. The ω-3 mice had significantly increased lipogenesis and ω-3:ω-6 ratio with no alteration in SCD products in the DMM joint compared to the non-operated joint.

SFA and ω-6 mice had decreased SCD and lipogenesis products in serum and synovial fluid from both limbs as compared to control mice (Supplementary Figure 5A–C). Despite the fact that ω-3 mice also exhibited reduced lipogenesis compared to control mice, the extent of decrease appeared to be smaller in ω-3 mice compared to that of the mice fed other HF diets. Furthermore, no alteration in SCD products in the synovial fluid from the DMM joint of the ω-3 mice was observed. All obese mice demonstrated increased serum D5D but decreased serum D6D products compared to control mice. The change in synovial fluid D5D and D6D in obese mice was diet dependent; however, a general trend toward decreased D5D and D6D products was found. As expected, ω-3 mice had significantly elevated ω-3: ω-6 ratio in both serum and synovial fluid relative to control mice.

For comparisons within obese mice, ω-3 PUFA rich HF diets notably up-regulated serum SCD and lipogenesis products versus both SFA or ω-6 PUFA rich HF diets (Supplementary Figure 5D–F). Furthermore, ω-3 mice showed decreased D5D products in the synovial fluid from the DMM joint compared to the SFA and ω-6 mice. Interestingly, increased D6D in the synovial fluid from the non-operated joint of ω-3 mice was observed compared to the SFA and ω-6 mice. When comparing ω-6 mice to SFA mice, ω-6 mice showed down-regulated SCD products, lipogenesis, and ω-3: ω-6 ratio in serum and the synovial fluid from the non-operated joint, although no difference in these signatures was found in the synovial fluid from the DMM joints between these two diets.

## Discussion

We showed that the lipid profiles in both serum and synovial fluid could be used as predictors for OA severity, synovitis, wound healing, and serum adipokine levels in an injury-induced OA model in obese mice, although individual lipids differ in their predicting capacity. We also observed that mice fed a HF diet rich in ω-3 PUFAs exhibited less perturbation in most lipid species and metabolic pathways (particularly in desaturase products) in the synovial fluid post-injury compared to the mice fed a HF diet rich in either SFAs or ω-6 PUFAs. Furthermore, ω-3 PUFAs generally demonstrated negative correlations with OA severity and ear wound area, while they positively correlated with adiponectin, an adipokine capable of promoting insulin sensitization and priming macrophages toward the M2 anti-inflammatory phenotype^26^. On the contrary, most ω-6 PUFAs exhibited positive correlations with markers of joint degeneration and systemic inflammation, such as leptin and resistin concentrations, and ear wound area. Moreover, we found that high serum ω-3: ω-6 PUFA ratios correlated robustly with decreased OA severity and serum inflammatory adipokine levels. These findings further corroborated previous studies, both ours and others, showing that ω-6 PUFAs play a role as pro-inflammatory mediators, whereas ω-3 PUFAs may act in an anti-inflammatory manner in obesity and OA in both human and animal models^27^.

An important and novel finding of the current study is that pentadecylic acid (C15:0), an odd-chain SFA, was negatively correlated with OA in both serum and synovial fluid levels, and also exhibited an inverse association with serum leptin concentration. It has been well-established that obesity promotes leptin resistance^28^. A recent report further demonstrates that leptin up-regulates IL-6 expression in human synovial fibroblasts, suggesting its role in joint inflammation^29^. Interestingly, we found that pentadecylic acid also showed a positive correlation with ear wound healing and serum adiponectin levels. These findings imply that pentadecylic acid may have different physiological effects on joint diseases, inflammation, and wound healing compared to even-chain SFAs such as palmitic and stearic acid. While most even-chain SFAs are generally considered to be pro-inflammatory in metabolic syndromes and cardiovascular diseases (CVD), due to their ability to induce dimerization and recruitment of Toll-like receptor 4 into lipid rafts^30^, recent studies have reported that odd-chain SFAs including pentadecylic acid and heptadecanoic acid (C17:0) demonstrate an inverse association with the risk of developing CVD and type II diabetes^31^,^32^. However, the mechanism underlying how odd-chain SFAs influence disease progression remains to be elucidated, although it has been suggested to involve the increased fluidity of the cell membrane by the presence of these odd-chain FAs^33^.

Interestingly, we also found that palmitoleic acid was negatively correlated with OA in serum and negatively associated with joint synovitis in synovial fluid if normalized concentrations were used for regression, implying its potential anti-inflammatory effect in our obese animal model. There is evidence suggesting that palmitoleic acid can improve insulin sensitivity in muscle cells by modulating the activation of macrophages^34^. However, the exact role of palmitoleic acid in inflammation and metabolic diseases is still unclear and controversial. In a clinical study investigating the association of erythrocyte membrane FAs with the concentration of C-reactive protein (CRP) in non-obese, non-diabetic men in Finland, the authors found that palmitoleic acid positively correlated with CRP^35^. On the contrary, Bernstein *et al*. reported that dietary supplementation of purified palmitoleic acid for 30 days was sufficient to reduce serum CRP, triglyceride, and low-density lipoprotein, but significantly increased high-density lipoprotein in adults with dyslipidemia, although this clinical study was performed in a smaller scale^36^. These discrepancies between the above-mentioned studies may arise from the obese status of the subjects as the effects of palmitoleic acid may act differently dependent on adiposity^37^.

We also reported the predictability of four plasmalogen derivates for inflammation, joint degeneration, and wound healing. Unlike ester-linked phospholipids, plasmalogens are ether-linked phospholipids characterized by the presence of a vinyl-ether linkage between the fatty acid chains and glycerol backbone. Plasmalogens often exist with the ether linkage at the *sn-1* position, while its *sn-2* position is occupied typically by PUFAs. The alkenyl groups from the ether linkage at the *sn-1* position are most commonly occupied by C16:0, C18:0, or C18:1 fatty alcohols^38^. In the current study, we found that levels of plasmalogens were significantly increased in the DMM joint compared to those in the contralateral non-operated joint, independent of units used. Furthermore, absolute concentrations of serum plasmalogen palmitic (dm16:0), plasmalogen stearic (dm18:0), and plasmalogen oleic (dm18:1n9) were positively associated with serum leptin levels, while normalized concentration of serum plasmalogen oleic positively correlated with ear wound area. Furthermore, normalized concentrations of synovial fluid plasmalogen palmitic, plasmalogen vaccenic (dm18:1n7), and plasmalogen oleic had positive associations with OA. Together, these findings imply that concentrations of various plasmalogen species were altered in OA, and plasmalogen derivates appear to be a predictor for cartilage degradation and inflammation in our obese mouse model. Our data are consistent with the results of a recent study showing that OA and rheumatoid arthritis patients had markedly increased plasmalogen levels in the synovial fluid as compared to healthy individuals, and the extent of increase in plasmalogen levels was disease-stage dependent^22^. Donovan *et al*. also reported that obesity increases serum plasmalogens^39^. In addition to being potential biomarkers for OA and obesity-associated inflammation, plasmalogens may be actually involved in the disease progression of OA. For example, phospholipids in the synovial fluid play an essential role in lubricating articular cartilage surfaces; thus, the change of the composition of phospholipids such as plasmalogens in the synovial fluid may impact lubrication efficiency, which may lead to degradation of cartilage^40^. Moreover, peroxisome-deficient animals demonstrated dysregulated biosynthesis of plasmalogens, and severe cartilage destruction^41^.

Independent of the concentration units used, the serum lipid profile more highly correlated with the synovial fluid lipid profile of the non-operated joint than that of the DMM-operated joint. This finding also indicates that serum lipid concentrations do not always reflect the lipid levels in the synovial fluid from the OA joint, and that injury and/or associated inflammation can significantly change lipid signatures in the joint environment. These changes may be partially due to the release of lipids from the damaged cells or increased permeability of inflamed synovium resulting from neoangiogenesis^42^. Furthermore, alteration of the lymphatic vessels may also contribute to the change in the synovial fluid composition in OA joints. For example, it has been demonstrated that osteoarthritic mice exhibited decreased mature lymphatic vessels, leading to decreased lymphatic drainage and impaired clearance of macromolecules^43^,^44^. Similarly, deceased densities of lymphatic vessels in the synovium of human OA patients has also been reported^45^. Another important factor affecting synovial fluid composition is the elevated production of inflammatory cytokines and the fragments of degraded cartilage matrix after injury, which also provides a possible explanation for our observation that the absolute concentrations of most lipid species in the synovial fluid from the OA joint were decreased compared to those in the normal joint fluid^46^,^47^.

Dysregulation of FA metabolism signatures, most notably in the SCD, D5D, and D6D pathways, were identified in SFA and ω-6 mice, whereas knee injury alone did not induce overall alterations of these pathways in synovial fluid for control mice. It is well established that SCD is responsible for indigenous biosynthesis of MUFAs including palmitoleic acid^48^. When compared to lean mice, all obese mice exhibited decreased SCD products, indicating reduced SCD activity in obese mice. The reduced SCD activity can be confirmed by the observation of low levels of palmitoleic acid in their serum and synovial fluid. This result supports the evidence that SFAs and PUFAs are involved in regulating SCD activity^49^. Interestingly, however, among the mice fed HF diets, ω-3 mice had relatively high concentrations of palmitoleic acid in both serum and synovial fluid of the DMM joint, suggesting higher SCD activity than SFA and ω-6 mice. This observation also implies that ω-3 and ω-6 PUFAs may have differential regulatory mechanisms on SCD expression or activity, but this inference requires further investigation.

The D5D and D6D are key enzymes in endogenous synthesizing of long chain (LC) ω-3 and ω-6 PUFAs from MUFAs. HF diet-induced obesity dysregulated the activity of these two enzymes in our mouse model, as D5D and D6D products were substantially altered in obese mice compared to those in control mice. This result is consistent with several animal and clinical studies showing that dietary fat content modifies desaturase activity, although it remains to be determined whether obesity up- or down-regulates the activities of these enzymes^50^. The activity of desaturase is often estimated by FA product and precursor ratio in any given tissue. However, such estimation does not exclude the LC PUFAs derived from diets (i.e. contributed exogenously), and may cause calculated activity to deviate from the actual activity of the enzyme. Therefore, precautions need to be taken for interpretation of the data when FA ratio is used as index for desaturase activity and when diets already contains high amount of LC ω-3 and ω-6 PUFAs.

In our current model, normalized concentrations of both serum and synovial fluid lipids exhibited stronger correlation with OA, synovitis and ear wound area compared with the absolute concentrations of the lipid species. For instance, 9 out of 34 synovial fluid lipids showed significant association with synovitis when the normalized concentration was applied as the unit; however, none of synovial fluid lipids correlated with synovitis if absolute concentration was used in the regression model. This distinction could potentially arise as a result of normalized concentration only accounting for the alteration of the FAs, while absolute concentration may be affected by the presence of a variety of other cytokines and metabolites, diluting the absolute concentration of lipid species in the synovial fluid and causing larger variability of this value. Therefore, we recommend that both absolute and normalized concentration of a lipid biomarker are reported as they may lead to distinct correlations with a given disease marker.

One potential limitation of the current study is that contralateral limbs were used as control for DMM surgery. Therefore, we cannot exclude any potential effect of the surgery such as altered gait and/or joint loading on the contralateral limbs^51^,^52^. However, our surgical outcomes are consistent to numerous studies demonstrating that arthritic changes were significantly more prominent and severe in the operated joints than contralateral and sham-operated joints^53^,^54^. Nevertheless, future studies may wish to use both non-surgical naïve and sham-operated limb as control groups for surgically-induced OA model, providing the most ideal control of the study.

In summary, our findings indicate that serum levels of ω-3 PUFAs were negatively correlated with OA severity and ear wound size, while most ω-6 PUFAs exhibited positive correlations with OA, impaired wound healing, and inflammatory adipokines. Furthermore, pentadecylic acid, an odd-chain SFAs, may have a protective role in OA and wound regeneration. This study extends our understanding of the links of FAs with OA, synovitis, and wound healing, and reports newly identified serum and synovial fluid FAs as predictive biomarkers of OA and wound repair in obesity.

## Additional Information

**How to cite this article**: Wu, C.-L. *et al*. Serum and synovial fluid lipidomic profiles predict obesity-associated osteoarthritis, synovitis, and wound repair. *Sci. Rep.* **7**, 44315; doi: 10.1038/srep44315 (2017).

**Publisher's note:** Springer Nature remains neutral with regard to jurisdictional claims in published maps and institutional affiliations.

## Supplementary Material

Supplementary Materials

## Figures and Tables

**Table 1:** Correlations between the absolute concentration (nMole/gram) of serum lipid species with OA, synovitis, and ear wound area.

		OA severity	Synovitis	Ear wound area
Predicator variables		*r*	*r*	*r*
**SFAs**	myristic acid	0.01	0.13	−0.36*
	pentadecylic acid	−0.45**	−0.16	−0.46**
	stearic acid	0.37*	0.40*	−0.01
**MUFAs**	myristoleic acid	0.08	−0.23	0.42*
	palmitoleic acid	−0.35*	−0.33	0.18
	erucic acid	−0.35*	−0.12	−1.97
	nervonic acid	−0.15	**−0.41**	−0.04
**ω-6 PUFAs**	linoleic acid	0.44**	0.41*	−0.10
	γ-linolenic acid	0.41*	0.36*	0.23
	eicosadienoic acid	0.38*	0.40*	−0.08
	arachidonic acid	0.39*	0.28	0.23
**ω-3 PUFAs**	α-linolenic acid	−0.14	0.11	−0.57**
	stearidonic acid	−0.47**	−0.19	−0.49**
	ETA	−0.58**	−0.37*	−0.38*
	EPA	−0.52**	−0.25	−0.46**
	ω-3: ω-6 ratio	−0.48**	−0.30 (p = 0.08)	−0.36*

ETA: eicosatetraenoic acid; EPA: eicosapentaenoic acid. *p < 0.05; **p < 0.01. None of plasmalogen correlates with OA, synovitis or wound score.

**Table 2:** Correlations between the absolute concentration (nMole/gram) of serum lipid species and serum adipokines.

	Predicator variables	Insulin	Leptin	Adiponectin	Resistin
		*r*	*r*	*r*	*r*
**SFAs**	pentadecylic acid	−0.17	−0.34*	0.35*	−0.27
	palmitic acid	0.46**	0.63**	−0.26	0.13
	stearic acid	0.65**	0.84*	−0.03	0.34*
**MUFAs**	oleic acid	0.23	0.23	−0.35*	−0.10
**ω-6 PUFAs**	linoleic acid	0.61**	0.84*	−0.12	0.31
	γ-linolenic acid	0.46*	0.78*	−0.34*	0.44**
	eicosadienoic acid	0.48*	0.72*	−0.15	0.22
	dihomo- γ-linolenic acid	0.52**	0.57**	−0.28	0.05
	arachidonic acid	0.66*	0.83**	−0.15	0.31
	adrenic acid	0.20	0.36*	−0.19	0.32
**ω-3 PUFAs**	α-linolenic acid	−0.18	−0.38*	0.18	−0.32
	stearidonic acid	−0.29	−0.67**	0.26	−0.33*
	ETA	−0.27	−0.61*	0.25	−0.38*
	EPA	−0.28	−0.56*	0.44**	−0.31
	DHA	−0.35*	−0.34*	0.37*	−0.11
**Plasmalogen derivatives**	dm16:0	0.01	0.36*	−0.30	0.08
	dm18:0	0.32	0.61**	−0.12	0.32
	dm18:1n9	0.28	0.50**	−2.42	0.23
	ω-3: ω-6 ratio	−0.34*	−0.59**	0.33*	−0.40*

ETA: eicosatetraenoic acid; EPA: eicosapentaenoic acid; DPA: all-cis-7,10,13,16,19-docosapentaenoic acid; DHA: docosahexaenoic acid; dm: dimethyl. *p < 0.05; **p < 0.01.

**Table 3:** Correlations between the absolute concentration (nMole/gram) of synovial fluid lipid species with OA and synovitis.

	Predicator variables	OA severity	Synovitis
		*r*	*r*
**SFAs**	myristic acid	−0.5*	−0.22
	pentadecylic acid	−0.58*	−0.22
	palmitic acid	−0.46*	−0.28
**MUFAs**	myristoleic acid	**−0.43**	−0.32
	palmitoleic acid	−0.43*	−0.28
**ω-6 PUFAs**	γ-linolenic acid	−0.41*	−0.27
	docosadienoic acid	0.44*	0.23
**ω-3 PUFAs**	stearidonic acid	−0.44*	−0.16
	ETA	−0.60**	−0.22
	EPA	−0.49*	−0.20
	DPA	−0.48*	−0.13
	DHA	−0.5*	−0.20
	ω-3: ω-6 ratio	−0.14	−0.02

ETA: eicosatetraenoic acid; EPA: eicosapentaenoic acid; DPA: all-cis-7,10,13,16,19-docosapentaenoic acid; DHA: docosahexaenoic acid; None of plasmalogen correlates with OA or synovitis.*p < 0.05; **p < 0.01.
