# Supplementary material for: Serum and synovial fluid lipidomic profiles predict obesity-associated osteoarthritis, synovitis, and wound repair
Source: Sci Rep. 2017 Mar 20;7:44315. doi: 10.1038/srep44315 (PMC5357837; doi:10.1038/srep44315)
Supplement: Supplementary Materials [file srep44315-s1.pdf]

Supplementary Information

Serum and synovial fluid lipidomic profiles predict obesity-associated osteoarthritis, synovitis, and wound repair

Chia-Lung Wu[1,2], Kelly A. Kimmerling[1,2], Dianne Little[3], Farshid Guilak[1,2,*]

[1]Department of Orthopaedic Surgery, Washington University, St. Louis MO, 63110
[2]Shriners Hospitals for Children – St. Louis, St. Louis MO, 63110
[3]Departments of Basic Medical Sciences and Biomedical Engineering, Purdue University, West Lafayette, IN, 47907

*Corresponding author:
Farshid Guilak, Ph.D.
Departments of Orthopaedic Surgery, Developmental Biology, and Biomedical Engineering
Co-Director, Center of Regenerative Medicine
Washington University, St. Louis
Campus Box 8233
4515 McKinley Ave.
St. Louis, MO 63110-1624
guilak@wustl.edu
Tel. 314-362-7239

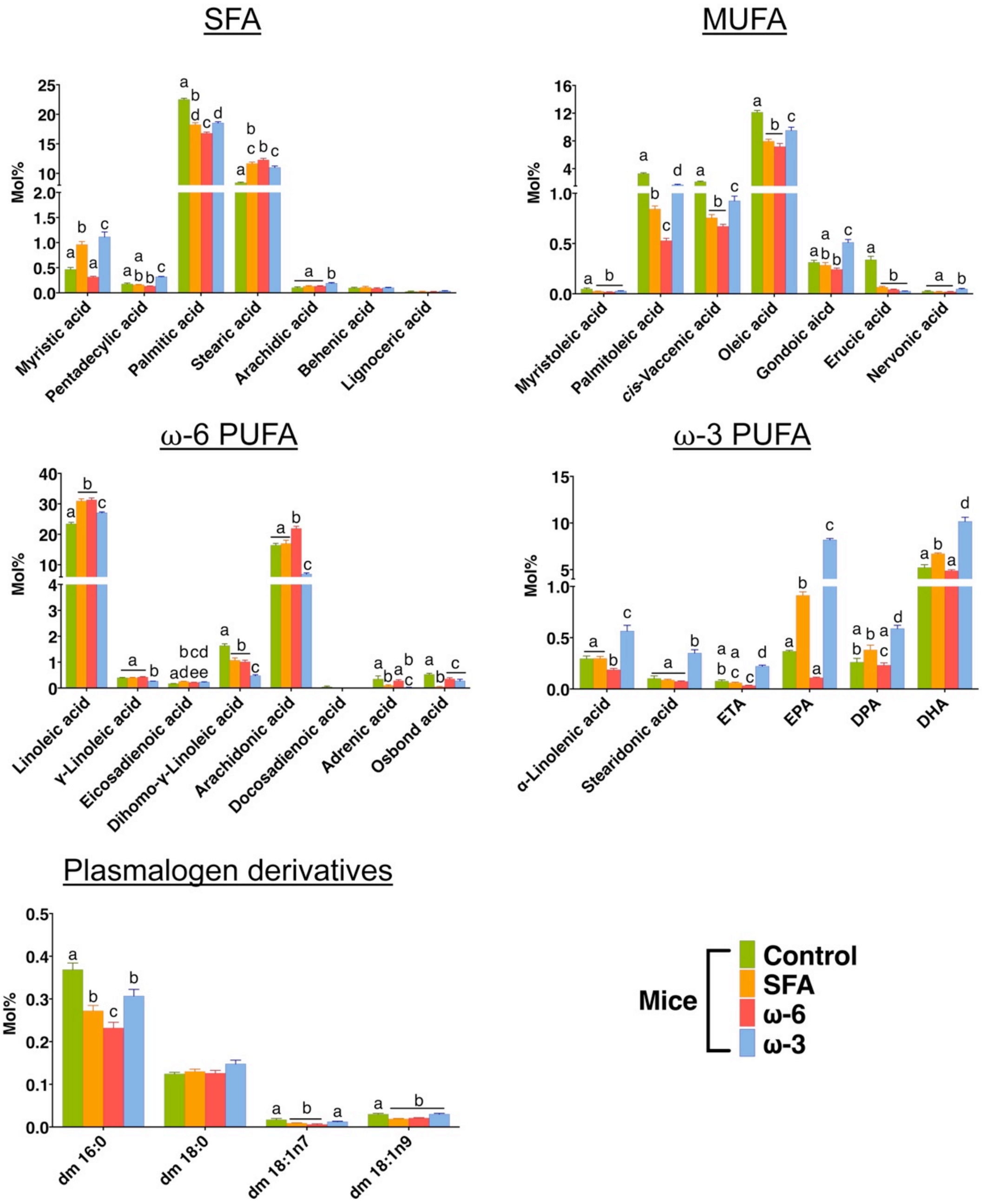

Supplementary Figure 1. Normalized concentrations of serum lipid species (Mol%). The ω-3 mice exhibited relatively low normalized concentration of ω-6 PUFAs, but significantly higher normalized concentration of ω-3 PUFAs. Furthermore, normalized concentration of serum DHA of ω-3 mice was significantly higher than that of the mice treated with other diets. One-way ANOVA with Tukey's post-hoc was performed to evaluate the effect of diet on each lipid concentration. Different letters are significantly different from each other ($p < 0.05$).

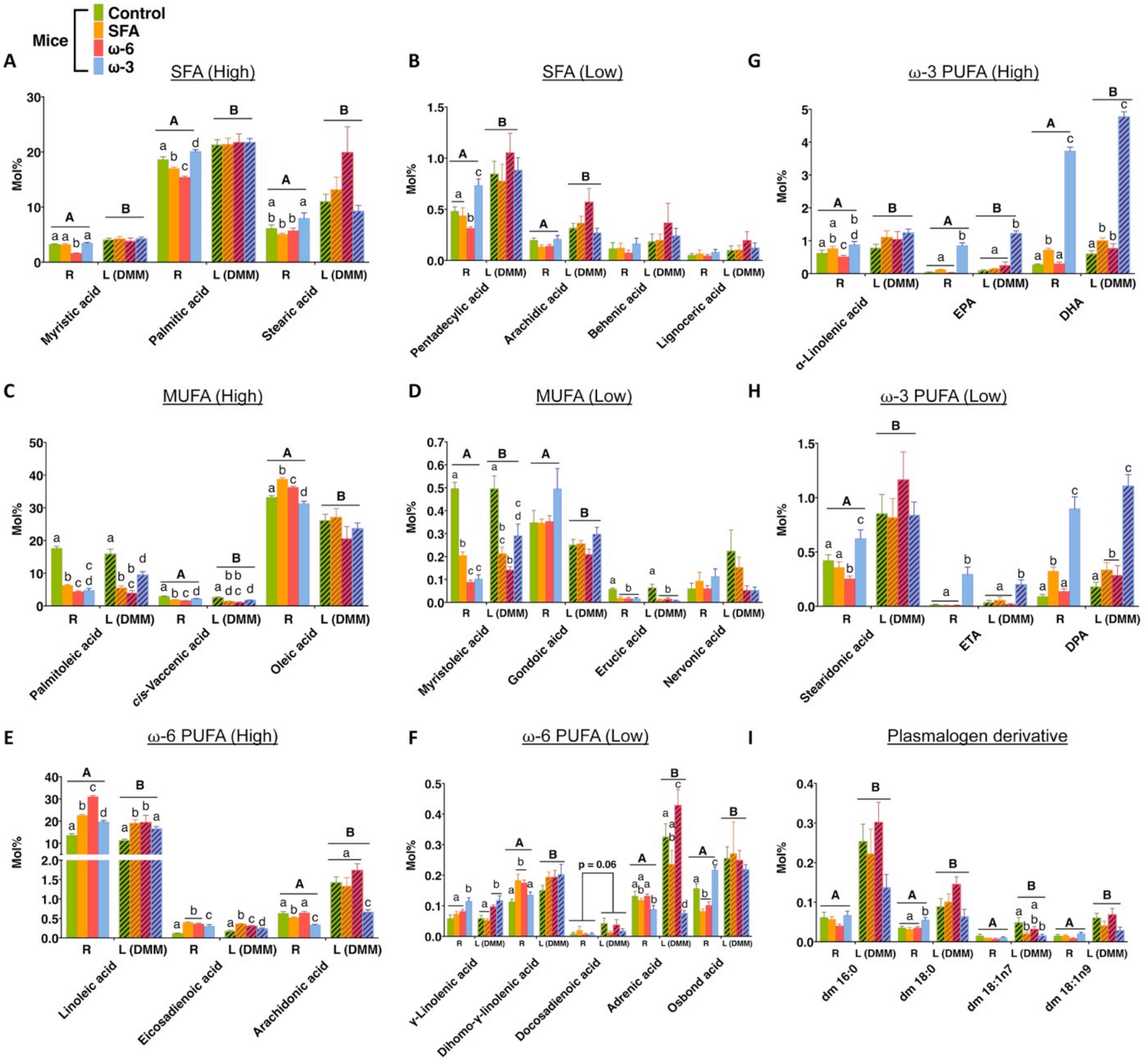

Supplementary Figure 2. Normalized concentrations of the lipid species in the synovial fluid from non-operated right (R) joint and from DMM-operated left (L) joint. (A), (C), (E) and (G) are FA plots with higher concentrations, while (B), (D), (F), and (H) are FAs plots with lower concentrations. The ω-3 mice had significantly higher normalized concentrations of most ω-3 PUFAs in synovial fluid from both non-operated and DMM joints. Two-way repeated measures ANOVA with Tukey's post-hoc was performed to evaluate the effect of diet and surgery on each lipid concentration. Different letters are significantly different from each other ($p < 0.05$).

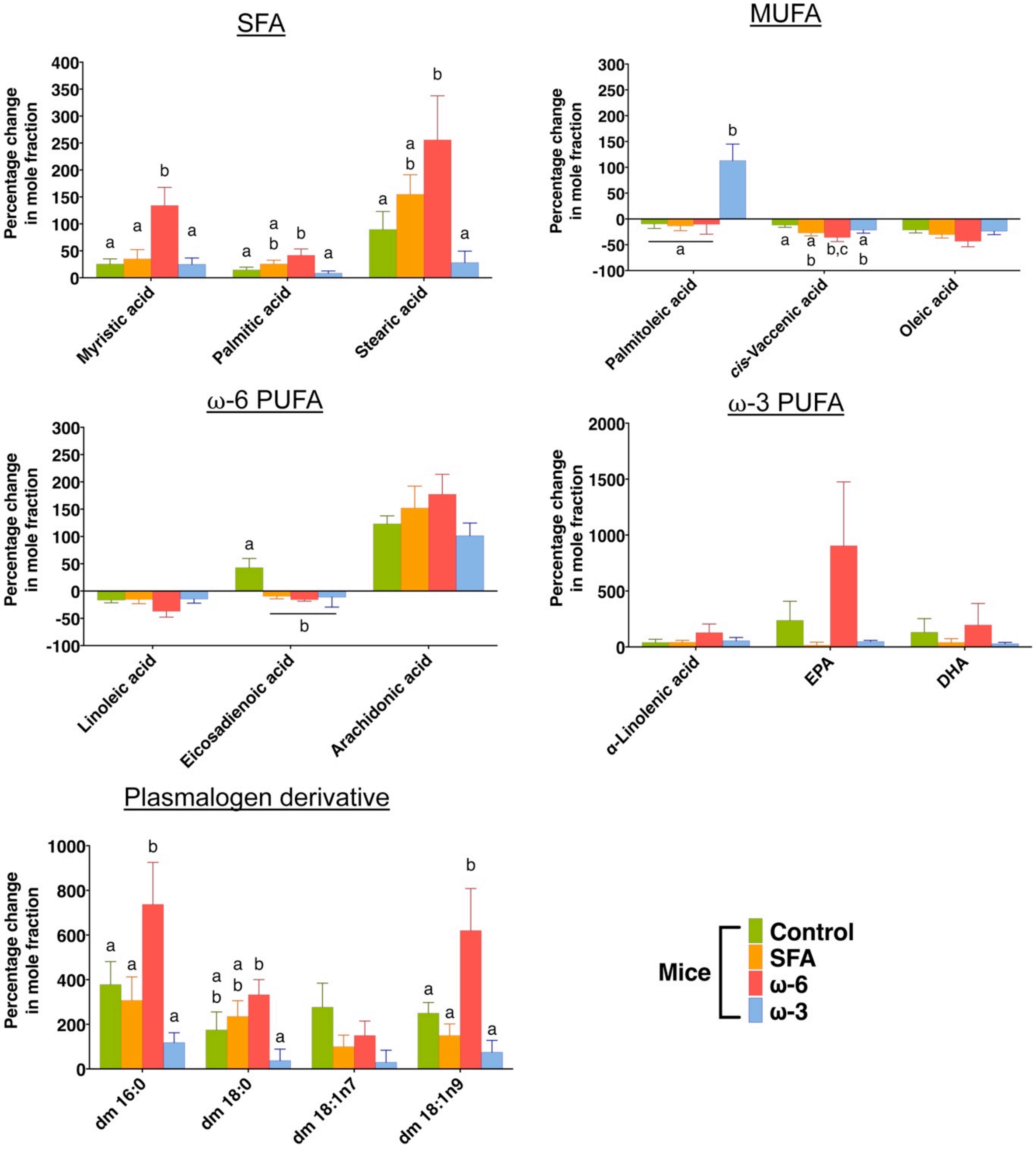

Supplementary Figure 3. The percentage change of mole fraction for major synovial FAs prior- and post-DMM surgery. In contrast to the absolute concentration, surgery significantly increased the normalized concentrations of most lipid species in synovial fluid in the operated joints. Furthermore, ω-6 mice exhibited substantial changes in the normalized concentration of synovial fluid FAs post-surgery, while ω-3 mice appeared to have fewer modifications in synovial fluid FA levels among the obese mice. One-way ANOVA with Tukey's post-hoc was performed to evaluate the effect of diet on each lipid concentration. Different letters are significantly different from each other ($p < 0.05$).

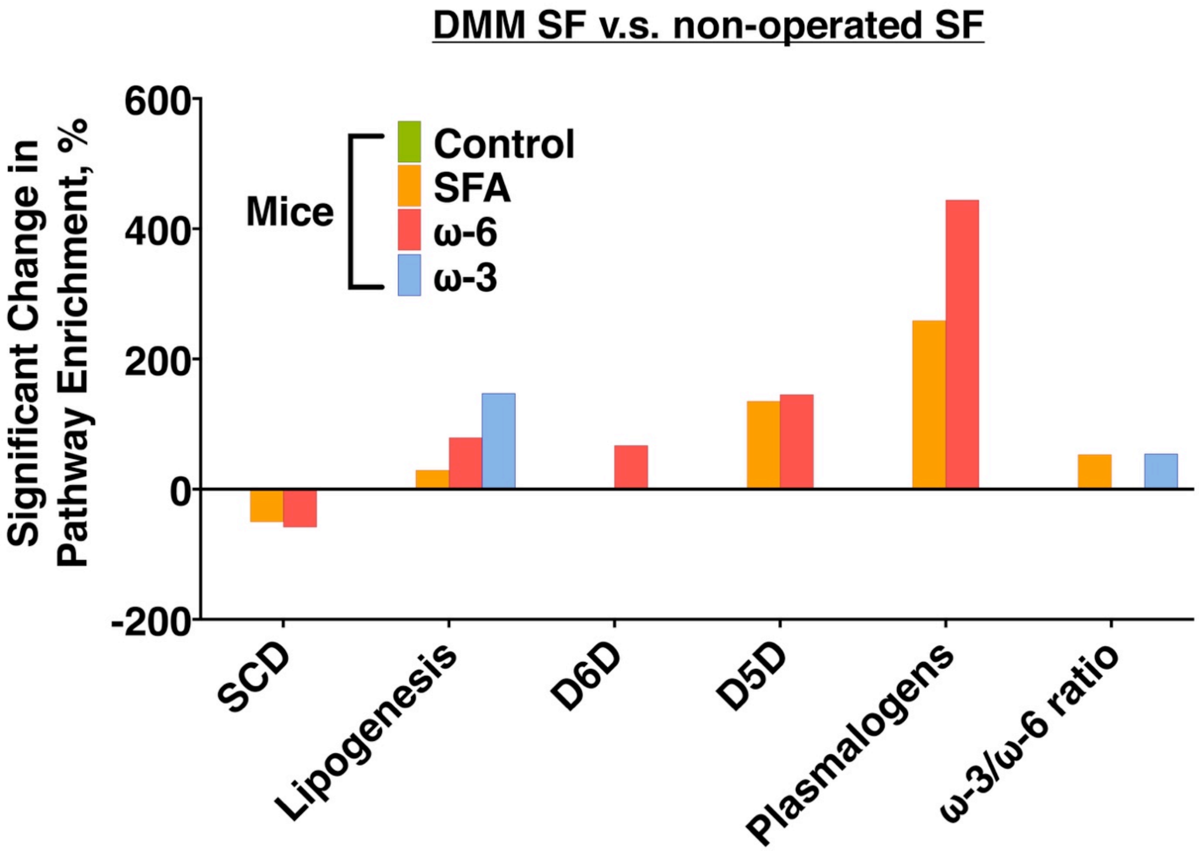

Supplementary Figure 4. The effect of the injury on the metabolic signatures in the synovial fluid within each diet group. There was no difference in FA metabolism signatures between DMM and non-operated limb SF in control mice. The SFA mice and ω-6 mice had increased lipogenesis, D5D products, and plasmalogens, but decreased SCD products in the SF from the DMM joint compared to those from the non-operated joint. The ω-3 mice had increased lipogenesis and ω-3:ω-6 ratio with no alteration in SCD products in the DMM joint compared to the non-operated joint. SF: synovial fluid; SCD: stearoyl-CoA desaturase; D5D: Δ-5 desaturase; D6D: Δ-6 desaturase.

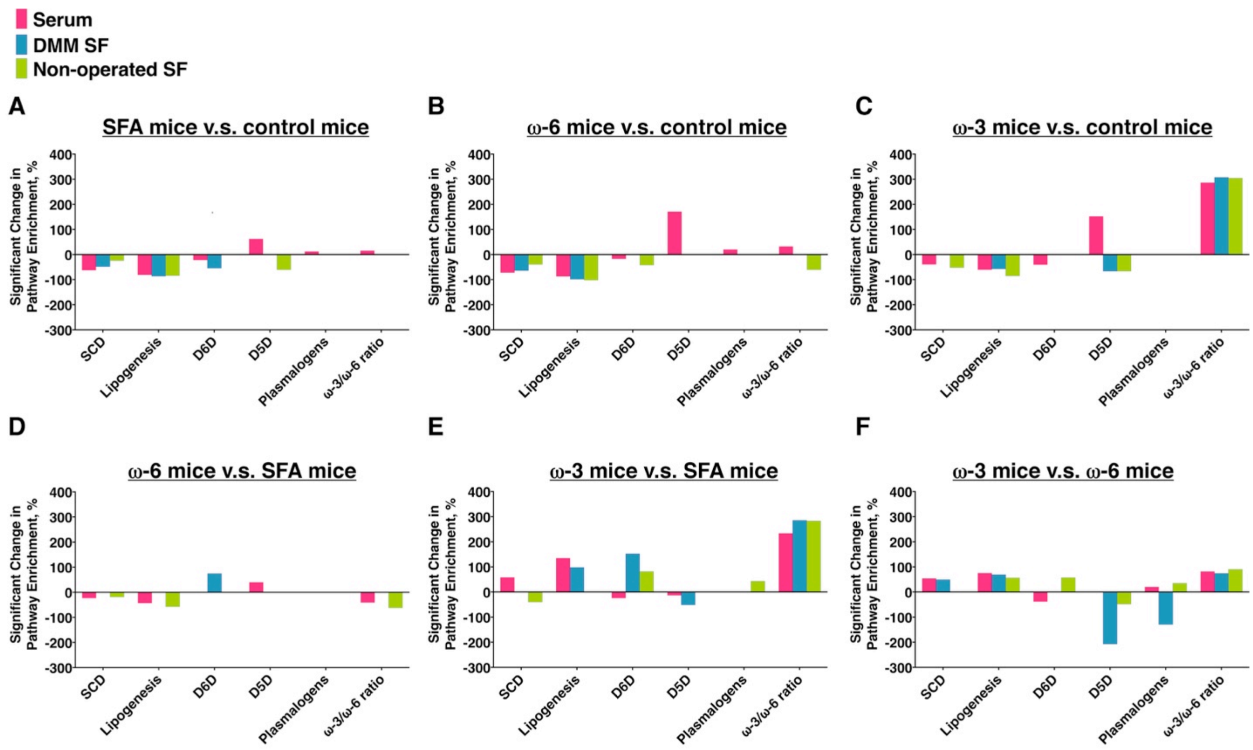

Supplementary Figure 5. The effect of HF diet rich in (A) SFA, (B) ω-6 PUFAs, and (C) ω-3 PUFAs on changes of FA metabolism signatures in serum and SF as compared to control mice. All obese mice demonstrated increased serum D5D but decreased serum D6D products compared to control mice. The change in SF D5D and D6D in obese mice was diet dependent, but a general trend toward decreased D5D and D6D products was found. (D-F) Comparisons of changes of FA metabolism signatures in serum and SF among obese mice. ω-3 mice up-regulated serum SCD and lipogenesis products versus did either SFA or ω-6 mice. The ω-3 mice also showed decreased D5D products in the SF from the DMM joint as compared those in SFA and ω-6 mice. When comparing ω-6 mice to SFA mice, ω-6 PUFAs down-regulated SCD products, lipogenesis, and ω-3:ω-6 ratio in serum and the SF from the non-operated joint, although no difference in these signatures was found in the SF from the DMM joints between these two diets. SF: synovial fluid; SCD: stearoyl-CoA desaturase; D5D: Δ-5 desaturase; D6D: Δ-6 desaturase.

Supplementary Table 1. Lipid species investigated in the current study

**SFAs**	myristic acid (14:0)
	pentadecylic acid (15:0)
	palmitic acid (16:0)
	stearic acid (18:0)
	arachidic acid (20:0)
	behenic acid (22:0)
	lignoceric acid (24:0)
**MUFAs**	myristoleic acid (14:1n5)
	palmitoleic acid (16:1n7)
	cis-vaccenic acid (18:1n7)
	oleic acid (18:1n9)
	gondoic aicd (20:1n9)
	erucic acid (20:3n9)
	nervonic acid (22:1n9)
**ω-6 PUFAs**	linoleic acid (18:2n6)
	γ-linolenic acid (18:3n6)
	eicosadienoic acid (20:2n6)
	dihomo- γ-linolenic acid (20:3n6)
	arachidonic acid (20:4n6)
	docosadienoic acid (22:2n6)
	adrenic acid (22:4n6)
	osbond acid (22:5n6)
**ω-3 PUFAs**	α-linolenic acid (18:3n3)
	stearidonic acid (18:4n3)
	ETA (20:4n3)
	EPA (20:5n3)
	DPA (22:5n3)

	DHA (22:6n3)
**Plasmalogen derivatives**	plasmalogen palmitic (dm16:0)
	plasmalogen stearic (dm18:0)
	plasmalogen vaccenic (dm18:1n7)
	plasmalogen oleic (dm18:1n9)

Osbond acid: all-*cis*-4,7,10,13,16-docosapentaenoic acid; ETA: eicosatetraenoic acid; EPA: eicosapentaenoic acid; DPA: all-cis-7,10,13,16,19-docosapentaenoic acid; DHA: docosahexaenoic acid; dm: dimethyl

Supplementary Table 2. Correlations between the normalized concentration (Mol%) of serum lipid species with OA, synovitis, and ear wound area

Predicator variables		OA severity<br>*r*	Synovitis<br>*r*	Ear wound area<br>*r*
**SFAs**	myristic acid	-0.16	0.01	**-0.43***
	pentadecylic acid	**-0.41**	-0.26	**-0.33***
	palmitic acid	-0.15	-0.26	**0.38***
	stearic acid	0.23	**0.39***	-0.24
	arachidic acid	**-0.34***	0.01	**-0.47****
**MUFAs**	myristoleic acid	0.08	-0.21	**0.35***
	palmitoleic acid	**-0.37***	**-0.39***	0.22
	cis-vaccenic acid	-0.28	**-0.33***	0.31
	gondoic aicd	**-0.44****	-0.23	-0.32
	erucic acid	-0.16	**-0.33***	**0.41***
	nervonic acid	**-0.39***	-0.18	-0.17
**ω-6 PUFAs**	linoleic acid	**0.45****	**0.43****	-0.23
	γ-linolenic acid	**0.43****	0.31	**0.42***
	eicosadienoic acid	0.18	0.26	**-0.37***
	dihomo- γ-linolenic acid	0.12	-0.09	**0.50****
	arachidonic acid	**0.40***	0.22	**0.38***
	docosadienoic acid	0.14	-0.04	**0.35***
	adrenic acid	0.30	0.05	**0.42***
**ω-3 PUFAs**	α-linolenic acid	-0.27	-0.08	**-0.48****
	stearidonic acid	**-0.40***	-0.21	**-0.41***
	ETA	**-0.55****	**-0.35***	**-0.42***
	EPA	**-0.51***	-0.25	**-0.47****
	DPA	**-0.41***	-0.20	-0.26
	DHA	**-0.42***	-0.26	**-0.41***
**Plasmalogen derivative**	dm18:1n9	-0.08	-0.23	**0.33***

ω-3 : ω-6 ratio	**-0.5****	-0.27	**-0.38***

ETA: eicosatetraenoic acid; EPA: eicosapentaenoic acid; DPA: all-cis-7,10,13,16,19-docosapentaenoic acid; DHA: docosahexaenoic acid; dm: dimethyl. *$p < 0.05$; ** $p < 0.01$

Supplementary Table 3. Correlations between normalized concentration (Mol%) of synovial fluid lipid species with OA and synovitis

Predicator variables		OA severity	Synovitis
		r	*r*
**SFAs**	stearic acid	0.35	**0.42***
	arachidic acid	0.37	**0.42***
	behenic acid	**0.47***	**0.42***
	lignoceric acid	0.38	**0.52***
**MUFAs**	myristoleic acid	-0.32	**-0.43***
	palmitoleic acid	**-0.41***	**-0.48***
**ω-6 PUFAs**	dihomo- γ-linolenic acid	0.22	**0.42***
	arachidonic acid	**0.50***	0.33
	docosadienoic acid	**0.61****	**0.43***
	adrenic acid	**0.60****	**0.43***
**ω-3 PUFAs**	ETA	**-0.60****	-0.21
	DHA	**-0.43***	-0.16
**Plasmalogen derivatives**	dm16:0	**0.45***	0.31
	dm18:1n7	**0.40***	0.17
	dm18:1n9	**0.52****	0.31
ω-3 : ω-6 ratio		-0.27	-0.007

ETA: eicosatetraenoic acid; DHA: docosahexaenoic acid; dm: dimethyl. *p < 0.05; ** p < 0.01

Supplementary Table 4. Correlations between absolute concentration (nMole/gram) of serum lipid species and their corresponding synovial fluid lipid species (nMole/gram) from right and left joints

Predicator variables		Left (DMM) joint *r*	Right joint *r*
**SFAs**	myristic acid	**0.46***	**0.52****
	pentadecylic acid	**0.67****	**0.42***
**MUFAs**	palmitoleic acid	**0.72****	**0.60****
	cis-vaccenic acid	**0.42***	0.24
**ω-6 PUFAs**	linoleic acid	0.16	**0.60****
	γ-linolenic acid	**0.44***	0.05
	eicosadienoic acid	0.30	**0.61****
	dihomo- γ-linolenic acid	0.23	0.26
	arachidonic acid	**0.49***	**0.61****
	adrenic acid	**0.52****	**0.71****
**ω-3 PUFAs**	α-linolenic acid	**0.52****	0.13
	stearidonic acid	**0.67****	**0.66****
	ETA	**0.79****	**0.53****
	EPA	**0.95****	**0.95****
	DPA	0.40	**0.44***
**Plasmalogen derivatives**	dm16:0	0.34	**0.41***
	dm18:1n9	0.22	**0.51***
ω-3 : ω-6 ratio		**0.81****	**0.95****

ETA: eicosatetraenoic acid; EPA: eicosapentaenoic acid; DPA: all-cis-7,10,13,16,19-docosapentaenoic acid; dm: dimethyl. *$p < 0.05$; ** $p < 0.01$

Supplementary Table 5. Correlations between normalized concentration (Mol%) of serum lipid species and their corresponding synovial fluid lipid species (Mol%) from right and left joints

Predicator variables		Left (DMM) joint<br>*r*	Right joint<br>*r*
**SFAs**	myristic acid	0.19	**0.60****
	pentadecylic acid	0.08	**0.70***
	palmitic acid	0.04	**0.46***
**MUFAs**	myristoleic acid	**0.41***	**0.70****
	palmitoleic acid	**0.90****	**0.87****
	cis-vaccenic acid	**0.83****	**0.91****
	oleic acid	0.17	**0.56****
	erucic acid	**0.76****	**0.81****
**ω-6 PUFAs**	linoleic acid	**0.50***	**0.82****
	eicosadienoic acid	**0.57****	**0.73****
	arachidonic acid	**0.67***	**0.76****
**ω-3 PUFAs**	stearidonic acid	0.02	**0.58****
	ETA	**0.77****	**0.77****
	EPA	**0.93****	**0.97****
	DPA	**0.81****	**0.70***
	DHA	**0.85****	**0.88****
ω-3 : ω-6 ratio		**0.81****	**0.95****

ETA: eicosatetraenoic acid; EPA: eicosapentaenoic acid; DPA: all-cis-7,10,13,16,19-docosapentaenoic acid; DHA: docosahexaenoic acid. *p < 0.05; ** p < 0.01
